# Immobilization of Urease onto Modified Egg Shell Membrane through Cross Linking

**DOI:** 10.52547/ibj.26.2.132

**Published:** 2021-11-27

**Authors:** Fatemeh Morovvat, Seyed Ziae Aldin Samsam Shariat, Maryam Davoudi, Dariush Norouzian

**Affiliations:** 1 Department of Clinical Biochemistry, Isfahan Pharmaceutical Sciences Research Center, School of Pharmacy and Pharmaceutical Sciences, Isfahan University of Medical Sciences, Isfahan, Iran;; 2 Pilot Nano-Biotechnology Department, Pasteur Institute of Iran, Tehran, Iran

**Keywords:** Egg shell, Immobilization, Polyethylenimine, Urease

## Abstract

**Background:**

Immobilization is an approach in industry to improve stability and reusability of urease. The efficiency of this technique depends on the type of membrane and the method of stabilization.

**Methods:**

The PEI-modified egg shell membrane was used to immobilize urease by absorption and glutaraldehyde cross-linking methods. The membranes were characterized by FTIR and AFM, and Nessler method was applied to measure the kinetic of the immobilized enzymes. Finally, the storage stability (6 °C for 21 days) and reusability (until enzyme activity reached to zero) of the immobilized enzymes were investigated.

**Results:**

Based on FTIR, three new peaks were observed in both the absorption- (at 1389.7, 1230.8, and 1074.2 cm^-1^) and the cross-linking (at 1615-1690, 1392.7, 1450 cm^-1^) immobilized enzymes. The surface roughness of the native membrane was altered after PEI treatment and enzyme immobilization. The optimal pH of cross-linking immobilized enzymes was shifted to a more neutral pH, while it was alkaline in adsorption-immobilized and free enzymes. The reaction time decreased in all immobilized enzymes (100 min for free enzyme *vs.* 60 and 30 min after immobilizing by adsorption and cross-linking methods, respectively). The optimal temperature for all enzymes was 70 °C and they had a higher K_m _and a lower V_max_ than free enzyme. The stability and reusability of urease were improved by both methods.

**Conclusion:**

Our findings propose these approaches as promising ways to enhance the urease efficiency for its applications in industries and medicines.

## INTRODUCTION

Urea is one of the most important metabolic toxic compound, and the removal of the excess level of this product is challenging^[^^1^]. Urease (EC:3.5.1.5) is a metal-dependent enzyme catalyzing the detoxification of urea to ammonia and carbon dioxide with high efficiency. Urease is widely found in nature and plays an important role in the circulation of nitrogen. This enzyme has recently found various applications in biotechnology industry, e.g. for the assessment and detoxification of urea in some fluids such as blood, alcoholic beverages, and environmental wastewaters^[^^2^^–^^4^]. For industrial purposes, urease is provided from various organisms, including plants, bacteria, fungi, and invertebrates^[^^5^^,^^6^].

Due to low price and reusability, the immobilized enzyme has been considered as one of the most significant strategies in industry, medicine, and biotechnology^[^^7^]. Enzyme immobilization refers to physically entrapment of an enzyme in a certain region to maintain enzyme catalytic activities^[^^8^]. To date, several methods, such as entrapment, adsorption, covalent binding, encapsulation, copolymerization, and cross-linking, have been applied for enzyme immobilization^[^^9^]. Cross-linked enzyme crystal is also an approach employed to immobilize the enzymes. High productivity and time saving are the main advantages of this procedure. Glutaraldehyde, a bi-functional reactive molecule, is mostly used as a cross-linking agent^[^^10^]. The cross-linking may also enhance the enzyme activity of multimeric enzymes even better than multi-subunit ones^[^^11^]. Another enzyme immobilization method is adsorption, which is carried out by the non-covalent interaction such as van der Waals forces, ionic interactions, and hydrogen bond formation between a carrier and an enzyme^[^^12^]. In absorption technology, the structure of the immobilized enzyme does not change but prevents its active sites from disturbing to maintain its normal activity^[^^13^]. There are a wide variety of available carrier compounds to immobilize enzymes. These compounds can be divided into both organic and inorganic origin^[^^12^]. Recently, egg shell, a natural membrane-like structure, has widely been applied for immobilization purposes. This structure consists of proteins as major constituents. Egg shell membrane has also a large surface area and a porous structure without any interrupting interaction with analytes. Due to such properties, it has been chosen as an appropriate supportive membrane for enzyme immobilization^[^^14^]. 

Until now, urease immobilization has been performed in various ways. For instance, it has been immobilized on nylon tubes, carboxymethyl cellulose, polyacrylamide, and gelatin^[^^15^]. A former study has shown that urease adsorption on hydroxyapatite results in the elevation of enzyme stability and resistance to proteolytic hydrolysis^[^^16^]. Another related study used tagged magnetic nanoparticle as a strong support material for immobilization of urease^[^^17^]. The present study was designed to immobilize jack bean urease onto the egg shell membrane by cross-linking using glutaraldehyde and absorption methods. A significant focus of this work was to compare the urease activity, urease stability, and enzyme kinetics in cross-linked enzyme crystal and absorption methods.

## MATERIALS AND METHODS

**Materials**

Jack bean urease (EC:3.5.1.5) was purchased from Merck (Germany). Glutaraldehyde and poly-ethyleneimine were obtained from Sigma (USA). All chemicals and reagents used were of the analytical grade. 

**Urease immobilization by absorption**

 Eggs were first broken, and their eggshell membrane was carefully removed. The membrane was washed with HPLC grade water, cut into pieces of 2 cm in diameter, immersed in water and stored in a refrigerator at 6 °C overnight. The membrane was then immersed in PEI 2% (w/v, pH 7) in darkness for two hours. PEI is a polymer containing primary, secondary, and tertiary amino groups to chemically react with various regions of the enzyme for immobilization^[^^18^]. The excess amount of PEI was removed by washing, and then the membrane was immersed in an enzyme solution in PB for 4 h. Afterwards, 100 mg of the lyophilized enzyme was dissolved in PB (pH 7), followed by centrifugation at 100 ×g (Eppendorf Centrifuge 5418R, USA) for 1 min. The supernatant was used as a free source of urease. Thus, 50 µl of this enzyme solution was added to 950 µl of PB. To remove the excess amount of uncoupled enzyme, the membrane was washed with the HPLC grade water.

**Urease immobilization by cross-linking**

The PEI-modified eggshell membrane was prepared as mentioned in previous section. After eliminating the excess amount of PEI by washing, the membrane was immersed in a solution of enzyme in a PB for 4 h. The absorbed enzyme on membrane was then immersed in 1% v/v of glutaraldehyde solution, followed by washing in PB. 

**Determination of urease activity**

 To assess the activity of the urease, a standard curve of ammonia (a product of urease enzyme) was originally constructed. The ammonia can be determined colorimetrically by nesslerization^[^^19^^]^. Various concentrations of ammonia, ranging from 50 to 400 µM, were made. In this way the standard curve was constructed. The activities of the free urease were determined as described previously^[^^19^]. This method was based on liberated ammonia. In brief, 100 mg of the lyophilized enzyme was dissolved in PB (pH 7) followed by centrifugation at 100 ×g for 1 min. The supernatant was used as a free source of urease. Enzyme solution (50 µl) was diluted with 950 µl of PB. Also, 50 µl of this enzyme solution was added to 450 µl of urea at a concentration of 250 mM, but the blank contained urea and PBS. They were incubated at 37 ºC for 30 min. Next, 2 ml of Nessler’s reagent was added to 500 µl of assay solution. Finally, the absorbance of the mixture was measured at 405 nm against blank.

**AFM analysis**

AFM is a method for evaluating the modification of the support materials for enzyme immobilization. This method allows studying the topology, adhesion, elasticity, association processes, dynamics, and other properties of the immobilized enzymes. 

**FTIR study of immobilized enzyme**

 All types of the membranes were studied by FTIR spectra in the range of 400–4,000 cm^-1^ using a Jasco FTIR spectrometer (PerkinElmer, USA).

**Time, temperature, and pH optimization for enzyme and substrate interaction**

 In order to optimize the time, the free and immobilized enzyme were incubated in the presence of urea solution (250 mM, pH 7) at 37 °C at different times ranged from 5 to 200 min. Eventually, the activity of the enzyme was observed by the generation of ammonia. For optimization of temperature, the urea solution subjected to the free and immobilized enzymes at a temperature ranging from 0 to 70 °C. The optimization of pH was performed by subjecting the substrate (at 250 mM, pH 4-9) to the free and immobilized enzyme, and its activity was evaluated. 

**Michaelis–Menten constant**

 The effect of substrate concentration on the enzyme activity was investigated. In this regard, the concentrations of 2.4 to 12 mM of the substrate were prepared and incubated with the free and immobilized enzymes under optimal conditions of time, temperature, and pH. To compute Lineweaver-Burk Plot, we plotted the enzyme activity to 1/V and 1/S. The gradient of this chart was K_m_/Vmax and Y-intercept was -1/Vmax. 

**Enzyme stability investigation**

 After the completion of the enzyme immobilization process using both the physical absorption and cross-linking methods, enzyme stability was measured. For this purpose, the membranes were stored at 0.02 M of PB (pH 7) in a refrigerator at 6 °C for 21 days. Thereafter, the enzyme activity was again determined. 

**Recycling the immobilized enzyme**

 The possibility of multiple use of the immobilized enzyme was determined. To this end, the enzyme was incubated in 250 mM of substrate solution (pH 7) at 37 °C for 30 min. This process was carried out repeatedly for a period of time until the reaction did not produce any ammonia.

**Statistical analysis**

 The results were plotted as mean ± standard deviation of three separate experiments for all experiments.

## RESULTS

**Characterization of membranes**

**Fig. 1:**
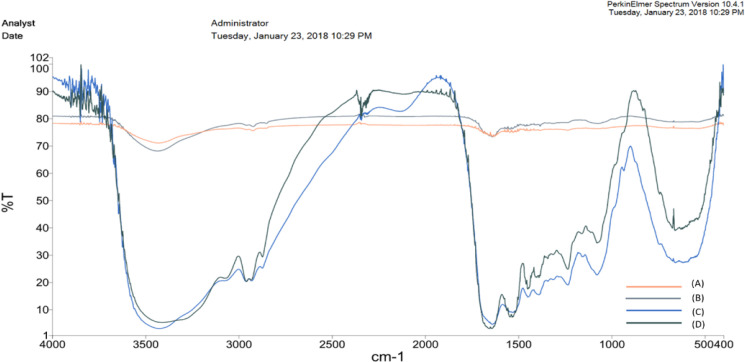
FTIR spectra of immobilized urease on the egg shell membrane. (A) Egg shell membrane; (B) the egg shell membrane immersed in PEI; (C) absorbed enzyme; (D) immobilized urease using glutaraldehyde

**Fig. 2:**
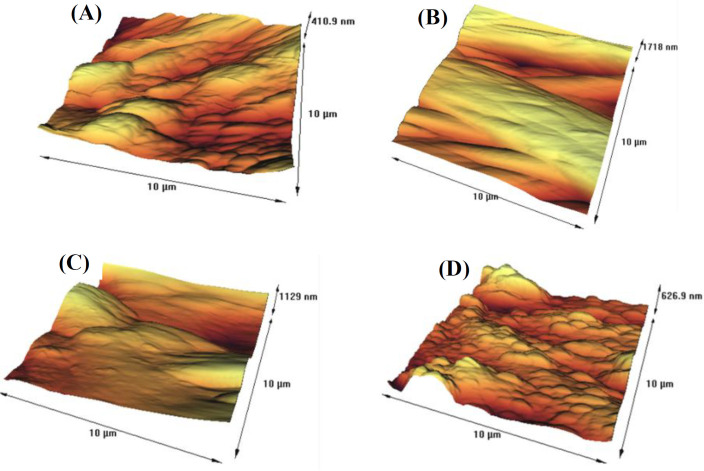
AFM topographic images of the egg shell membranes. (A) Clean egg shell membrane with the roughness of 69.869 nm; (B) the egg shell membrane immersed in PEI with the roughness of 374.1 nm; (C) an absorbed enzyme with the roughness of 197.73 nm; (D) a cross-linked enzyme with the roughness of 100.34 nm

The FTIR study of the egg shell membrane and immobilized enzymes was presented. As indicated in Figure 1, there was no sharp peak for egg shell membrane. However, some peaks were observed for the egg shell membrane incubated with PEI, and the peaks were located at around 2923.6, 1383.5, 1075.7, and 986.05 cm^-1^. Moreover, there were some new peaks between 1,000 and 1,600 cm^-1^ for the adsorbed and cross-linked enzyme. After immobilizing by adsorption method, three new peaks were appeared at 1389.7, 1230.8, and 1074.2 cm^-1^. These bonds indicated that urease has been successfully immobilized on membrane using C-N amide bonds^[^^20^^]^. After immobilizing by cross-linking method, three new peaks were observed at 1615-1690, 1392.7, and 1450 cm^-1^, which were related to the formation of imine bonds between urease and glutaraldehyde. In order to investigate the microstructure of the egg shell membrane with and without the immobilized urease using absorption and cross-linked methods, we conducted AFM. As shown in Figure 2, the roughness of the egg shell membrane was 69.869 nm, and surface of the membrane had a special structure without any aggregation; however, the membrane immersed in PEI increased the membrane roughness to 374.1 nm. The enzyme immobilization using absorption reduced the roughness of the egg shell membrane from 374.1 to 197.73 nm. Similarly, cross-linking immobilization decreased the roughness to 100.34 nm. 

**Characterization of immobilized and free enzymes**

**Fig. 3:**
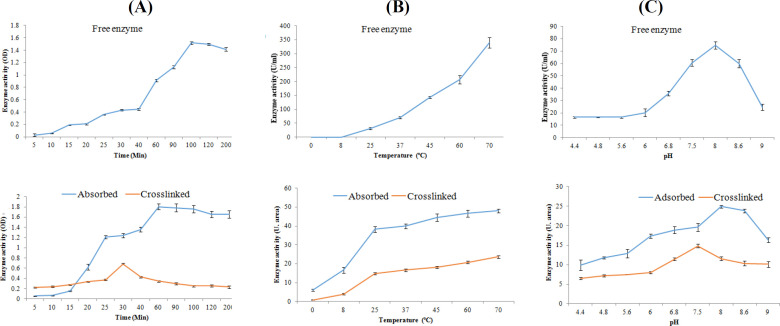
Kinetic study of free and immobilized enzymes

**Fig. 4:**
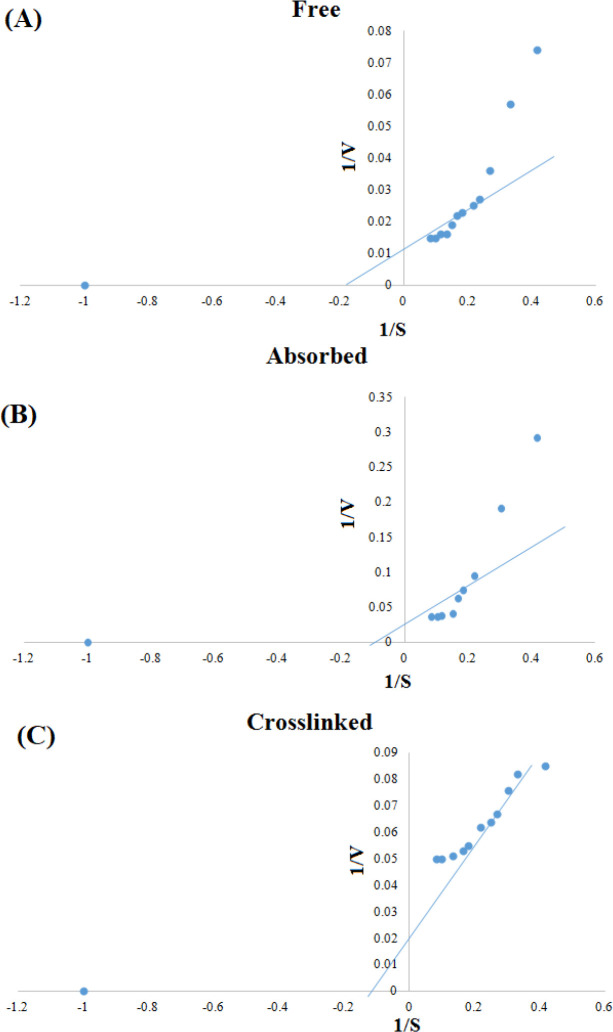
Profiles for enzyme optimization. The conditions of enzyme activity were optimized in term of (A) time, (B) temperature, and (C) pH

**Fig. 5:**
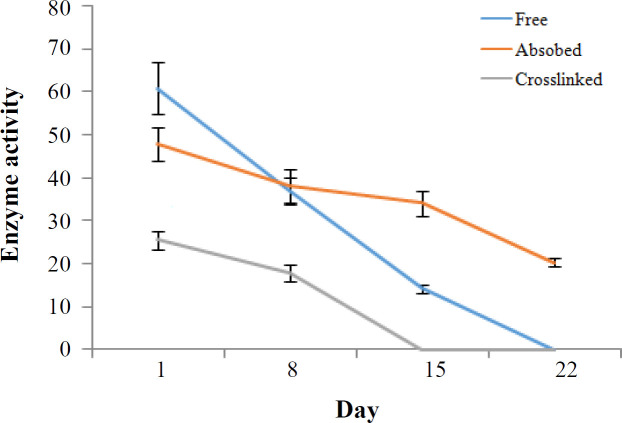
Enzyme stability analysis. The activities of free and immobilized enzymes were measured in a period of 22 days

**Fig. 6:**
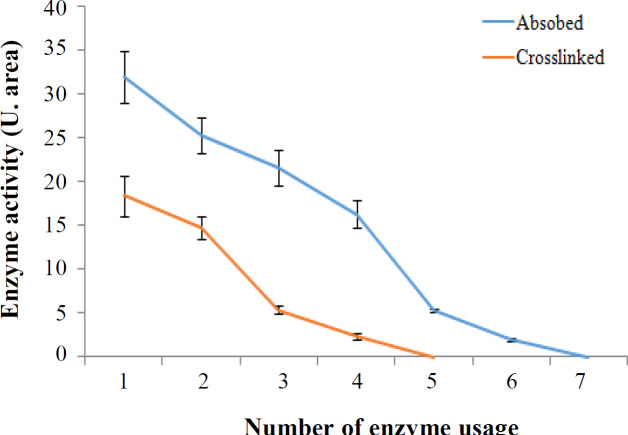
Reusability of immobilized enzyme. The activities of free and immobilized enzymes were evaluated after several using

 To optimize the time of incubation, we analyzed the samples at different incubation times (5-200 min). As presented in Figure 3A, the activity of the free enzyme after 60 min significantly increased, although the activity of the adsorbed enzyme enhanced faster (at 20 min) compared to that of the free enzyme in a similar manner. However, the activity of the cross-linked enzyme exhibited a different pattern and was optimized in the 30^th^ min. In other words, in time of sooner and later than 30 min, the enzyme had no different activity. Regarding the effect of temperature, the enzyme activity was significantly higher at the temperature above 37 °C. Moreover, the optimum temperature was observed to be 70 °C for the free enzyme. However, the activity of the immobilized enzyme did not change significantly with increasing temperature up to 70 °C (Fig. 3B). The effect of various pHs (from 4 to 9) on enzyme activity at the concentration of 250 mM urea demonstrated that the optimum pH for the free enzyme was 8. Both absorbed and cross-linked enzymes were not affected significantly by increasing pH (Fig. 3C). Kinetic analysis was performed using the Michaelis-Menten equation. A Lineweaver-Burk plot was drawn by inverting the concentrations of substrate and reaction velocity. As described previously, the K_m _value calculated from this equation is the evaluation of the affinity of the enzymes toward its substrates, with lower K_m_ value, indicating the higher affinity of the enzymes toward its substrates^[^^21^]. Based on the Figure 4, the K_m_ and V_max_ values for the free enzyme were found to be 6.25 mM and 100 U/ml, respectively. These values were 12.5 mM and 50 U/ml for the absorbed enzyme and 8.33 mM and 50 U/ml for the cross-linked enzyme, respectively. To measure the stability of the free and immobilized enzymes, we measured the enzyme stability from day 1 to day 22. As illustrated in Figure 5, enzyme activity for all types reduced over time. However, activity of the absorbed enzyme was significantly more than those of the free and cross-linked enzymes in days 15 and 22. Further analysis on the number of immobilized enzyme usage displayed that the cross-linked enzyme expired after four times of usage, but the activity of the absorbed enzyme maintained up to six times (Fig. 6). 

## DISCUSSION

Enzyme immobilization is a well-known method for improving the performance of important commercial enzymes. This method has advantages such as ease of synthesis, cost-effectiveness and increased efficiency of stabilized enzymes in terms of stability, reusability, reaction conditions (ex, pH, temperature, time of reaction, and substrate specificity) and storage period^[^^22^^–^^25^]. Over the past decades, immobilization technology has been expanded rapidly and has become increasingly conceptualized. Furthermore, various immobilization methods have been introduced for different types of enzymes. The type of matrix for enzyme immobilization is a challenging issue. Also, the cost-effectiveness of the reaction process is another concern for enzyme immobilization^[^^23^^,^^24^].

The present study used physical absorption and cross-linking on the egg shell membrane. Based on previous studies working on adsorption method, the enzymes could attach to a matrix through some interactions such as van der Waals and hydrogen bond. However, these interactions are weak and may allow the enzyme to leak from the membrane^[^^26^^–^^28^]. In addition to the adsorption method, the cross-linking method was also used in this study because it usually forms stronger interactions between enzymes and membranes than the absorption method. This way is often used in conjunction with other methods to enhance their efficiency and prevent the enzyme from leaking under industrial conditions. This method can also form an intramolecular cross-link and stabilize the protein. However, the use of this method has a major drawback because glutaraldehyde can form some intramolecular bonds in the structure of enzymes. These unwanted bonds may affect enzyme function by changing the conformation and active site of the enzyme^[^^29^^,^^30^]. To select the stabilization matrix, egg shell was considered as a natural organic membrane for the stabilization of the urease enzyme. The egg shell membrane was chosen because it consists of fibers with lateral joints, which make a very large surface area and provide a matrix to load large quantities of enzymes. Moreover, the egg shell membrane is non-toxic, water insoluble and highly resistant to organic solvents. Nonetheless, the low loading capacity of egg shell membranes has limited their application in biotech industry. Since this membrane is potentially suitable for the stabilization process, efforts have been made to adopt strategies to increase the enzyme loading capacity. The urease enzyme has an isoelectric point of 4.9-5.5, and above this pH, the urease has negative charge, enabling well absorption on positive charge surfaces of the PEI membrane. In the present study, this chemical agent was used for making positive charge on the egg shell membrane, which improved the attachment of the enzyme to the membrane^[^^31^]. 

After immobilization of the urease on membranes, the surface of the membranes was characterized using FTIR and AFM microscopy. Firstly, the FTIR results showed that the treatment of the egg shell membrane with PEI produced four new peaks at 2923.66, 1383.5, 1075.7 and 986.5 cm^-1^. The peaks were related to stretching of CH in alkenes, bonding of CH alkanes, stretching of CN of primary and secondary aliphatic amines, and N-H wag of primary and secondary amines, respectively. Similarly, after the immobilization of the urease using the physical absorption method, three new FTIR peaks were obtained at 1389.7, 1230.8, and 1074.2 cm^-1^. The FTIR peaks were related to CN extending the amines of aromatics, CN stretching the amine groups of aliphatic amines, and CN stretching of the primary and secondary aliphatic amines, respectively. Moreover, after the immobilization of the urease by cross-linking method, three new peaks of 1615-1690, 1392.7, and 1450 cm^-1^ were emerged, which were related to the stretching of C=N, CH bending of aldehydes ,and CH bending of alkanes, respectively. All these bonds, similar to those in D’Souza *et al.*’s^[^^20^] study are the result of the enzyme linked to glutaraldehyde in the stabilization process. The AFM-based microscopic study was the second method of determining surface membrane characteristics. AFM is the only microscopic technique capable of detecting the status of bio-molecules at the surface of a molecule and is widely used for biosensor production studies. Accordingly, in the present study, the topographic condition and surface roughness level of the membranes were measured before and after immobilization using an AFM microscope. The results showed that the egg shell surface alone had high roughness values of 69.89 nm, and there was a special structure without any aggregation, suggesting that the membrane was consisted of various protein fibers linked to each other^[^^32^]. The PEI treatment of egg shell membrane increased the roughness level (374.1 nm), since this chemical substance can alter the membrane surface. The AFM image confirmed the enzyme immobilization using both physical absorption and cross-linking. Based on the results, it seems that the stabilization of the enzyme by both physical adsorption and cross-linking on the PEI-treated egg membrane reduced the degree of membrane roughness (197.73 and 100.34 nm, respectively; Fig. 2). This is probably due to the fact that the enzyme is stabilized in the pores of the membrane and reduced the degree of roughness by filling these pores. Additionally, the cross-linking method reduced the roughness more than physical absorption method. 

In order to determine the optimal conditions of the enzymatic reaction, a kinetic study was performed on free and immobilized enzymes under different reaction conditions in term of pH, time, and temperature. In general, following the elevation of temperature, the enzyme activity first increased until it reached the optimum limit and then gradually decreased. The reason for this reduction is that with increasing temperature, the kinetic energy of the molecules raises, which results in increased movement and probability of molecular collisions^[^^33^]. However, when the energy exceeds a certain level, hydrophobic and hydrogen bindings are disrupted, leading to the reduced activity and, consequently, to the enzyme denaturation at higher temperatures. One of the goals of enzyme immobilization is to increase the enzyme temperature stability through the attachment to the desired matrix so that the enzyme can absorb more heat before it becomes denatured. Thus, in this study, the activity of the free and immobilized enzymes was measured at various temperatures (from 0 to 70 °C). The results showed that the optimum temperature for the aforesaid enzymes was 70 °C, and there was no significant difference between the activity of the free and immobilized enzymes in terms of optimal reaction temperature. Overall, the results showed that the temperature profile of the enzyme immobilization was slightly broader than that of the free one. The reason is that the free enzyme solution did not have any activity at 0 °C, whereas the immobilized enzyme was active in both physical absorption and cross-linking. Another important kinetic parameter affecting the activity of the enzyme is reaction of pH. A previous study has shown that stabilization of an enzyme on the matrix results in changes in its optimum pH by altering the structure and loading of the enzyme^[^^34^]. One of the enzyme immobilization objectives is the production of enzymes with high stability in a wide range of pH. The optimum pH of the free and the physically adsorbed enzyme was 8. However, this value was 7.5 for cross-linked enzyme. In a study by Nilsson* et al.*^[^^35^^]^, it was found that pH values in the small medium adjacent to the enzyme molecule may change based on surface changes, roots in the solid matrix, and nature of enzyme bonds, thereby changing the optimal pH of the enzyme. The concentration of the product (ammonia) in a small environment surrounding the enzyme is important because it easily affects reaction pH. Accordingly, the reduction of optimal pH for the egg shell cross-linked enzyme was more likely to be prevented owing to the release of the product. As a result of this phenomenon, the product (ammonia) around the enzyme was surrounded by a small environment surrounding the enzyme stabilized, resulting in a more alkaline pH than the total solution. 

At the end of the study, the optimal time for the free and immobilized enzymes was determined at a time interval between 5 and 200 min. The results showed that the optimum times for the enzyme-reactions were 100, 60, and 30 min for the free enzyme, the adsorption- and cross-linking immobilized enzyme, respectively. The comparison of these results with the data obtained from K_m_ and V_max_ showed that the processes used for immobilization may have limited the access of the substrate to the active site of the enzyme by creating a physical barrier, which increased K_m_ and decreased V_max_ in the immobilized enzymes compared to free enzyme^[^^36^]. However, reducing the optimal reaction time in a variety of immobilized enzyme indicates that the immobilization processes more likely affect the active site of enzymes. In other words, in many cases, upon the enzyme immobilization, the reaction time would be reduced when compared to the free enzyme. In addition, enzyme activity was more reduced by cross-linking than by physical absorption.

Kinetic parameters such as K_m_ and V_max_ were also calculated since immobilization matrices are often due to spatial inhibition, preventing the free distribution of the substrate among enzyme molecules. Thus, the stabilization process usually causes a change in K_m_ of enzymatic reactions. In this study, after the immobilization of the enzyme, K_m_ and V_max_ were calculated. Based on our results, K_m_ of the enzymes increased compared to that of the free enzyme. Similar changes have been reported in K_m_ values for the enzyme urease after immobilization on chitosan, gelatin and diethylaminoethyl cellulose matrices in other studies^[^^37^^,^^38^]. In this study, V_max_ value for the immobilized enzymes was 50 U/ml, which was less than the value achieved for the free enzyme (100 U/ml). In general, increasing K_m_ and decreasing V_max_ in the immobilized enzymes implies low availability of the substrate to the active site of the enzyme due to chemical bonding, release restriction, and encapsulation of enzyme molecules in the matrix, as reported in previous studies^[^^37^^,^^38^]. 

To industrialize enzymes, it is important to monitor the enzyme stability according to various parameters such as temperature, pH, reuse, storage stability, etc. In this study, the stability of the enzymes was investigated only in terms of storage conditions. For this purpose, the membranes were kept in a buffer of 0.02 M of PB with pH 7 in a refrigerator at 6 °C for 21 days. The results showed that the physical absorption of the enzyme on the egg shell membrane retained about 50% of its activity after 21 days. However, the total enzyme activity was lost 21 days after immobilizing by cross-linking, and the free initial enzyme remained active for only 15 days. In general, these results show that the physical absorption immobilization method, in contrast to the cross-linking technique, results in longer stability. In a study by Tembe *et al.*^[^^39^], it was found that enzyme immobilization was maintained on the egg membrane using the surface absorption method after two months in buffer at room temperature.

Since free enzymes are often dissolved in the reaction mixture, it is not practicable to retrieve and reuse them. As a result, maintaining enzymes in the process of immobilization and their reusing are important criteria that require to be considered in the process of enzyme immobilization. In this regard, in order to evaluate the possibility of enzyme usage, the enzyme fixed on the membranes and the free enzyme were incubated in 0.25 M of urea solution at pH 7 at 37 °C for 30 min. This procedure was performed repeatedly and continued until the reaction between the enzyme and the substrate did not produce any other product. The results showed that although the free enzyme was only usable once, the fixed enzyme was reusable for six times by adsorption and four times by cross-linking.

Taken together, the results of the present study revealed that the modification of egg shell membrane with PEI improved the rate of enzyme immobilization through changing the degree of roughness and the functional groups of the membrane surface. This study also showed that although both adsorption and cross-linking methods increased the stability and reusability of urease, the enzyme activity in cross-linking method was less than the absorption, which is probably due to the formation of intramolecular bonds in the structure of the enzyme by glutaraldehyde. We also optimized the enzymatic reaction of all immobilized ureases under different conditions (time, temperature, and pH). Based on data, the optimal pH of cross-linking immobilized enzymes was shifted to a more neutral pH; however, it was alkaline for adsorption-immobilized and free enzymes. While both immobilization methods reduced the reaction time (100 min for free enzyme *vs.* 60 and 30 min after immobilizing by adsorption and cross-linking methods, respectively), their optimal temperature was similar to that of free urease. Further studies are still needed to improve the efficiency and effectiveness of the proposed stabilization methods, but this pilot study has provided a promising outlook for achieving this goal in future. 

## DECLARATIONS

### Acknowledgements

We express our deepest gratitude to all those who helped us in Isfahan Pharmaceutical Sciences Research Center and School of Pharmacy and Pharmaceutical Sciences, Isfahan, Iran.

### Ethical statement

Not applicable. 

### Data availability

The analyzed data sets generated during the study are available from the corresponding author on reasonable request.

### Author contributions

FM, carried out the experiments; SZSS, critically revised the manuscript; MD, helped in analyzing the data; ND designed the project and analyzed the data.

### Conflict of interest

None declared.

### Funding/support

This study was the result of a research project approved and financially supported by Isfahan University of Medical Sciences (IUMS; contract number: 396387).
